# Neonatal brain abscess development following fetal scalp electrode placement: a rare complication

**DOI:** 10.1007/s00381-021-05150-7

**Published:** 2021-04-06

**Authors:** T. Fick, P. A. Woerdeman

**Affiliations:** Department of Neurosurgery, University Medical Center Utrecht, Wilhelmina Children’s Hospital, Lundlaan 6, 3584 EA Utrecht, Netherlands

**Keywords:** Neonatal, Brain abscess, Fetal scalp electrode

## Abstract

A fetal scalp electrode (FSE) is a frequently used investigation during labor. However, it is an invasive procedure which can lead to complications. Our patient developed a very large brain abscess after initial superficial infection of the skin site due to an FSE. The patient was admitted to the hospital after an asymmetric growth of the skull was noticed with no further signs of clinical illness. MRI showed a very large brain abscess which was aspirated and treated with antibiotics for 10 weeks. A 2-year follow-up showed only a slight developmental delay in gross motor skills. Only once before a similar case has been described at which the patient developed a brain abscess after superficial infection of the scalp following an FSE. In both cases, the brain abscess was noticed due to an asymmetric growth of the skull without any further signs of clinical illness. A brain abscess has a high mortality and morbidity rate, and early diagnosis is vital for the optimal outcome. We therefore recommend to organize an out-patient clinical follow-up for every infant with a superficial infection of the skin site after placement of an FSE.

## Introduction

Cardiotocography (CTG) is a technique to monitor the fetal heart rate and uterine contractions during labor. The indication is broad and can variate between countries [1]. CTG can be done externally by using two transducers on the mothers’ abdomen and internally by using an electronic transducer connected to the fetal scalp by an electrode. A fetal scalp electrode (FSE) accounts for a more accurate measurement and is less affected by movement compared to external monitoring and is therefore frequently used. In 22% of labors in clinical centers in the USA, an FSE is used [2]. However, this is an invasive procedure and can lead to complications. It is associated with a 1.3% rate of scalp ulcers and has an increased risk of developing a cephalohematoma and neonatal sepsis [3]. Scalp abscess development following FSE is well described and is present in up to 4.5% of the cases [4–6]. Although rare, more serious complications, such as cranial osteomyelitis and epidural abscesses, have been described [7, 8]. We present a case of an 8-week old infant with a very large brain abscess due to placement of an FSE.

## Case

**Fig. 1:**
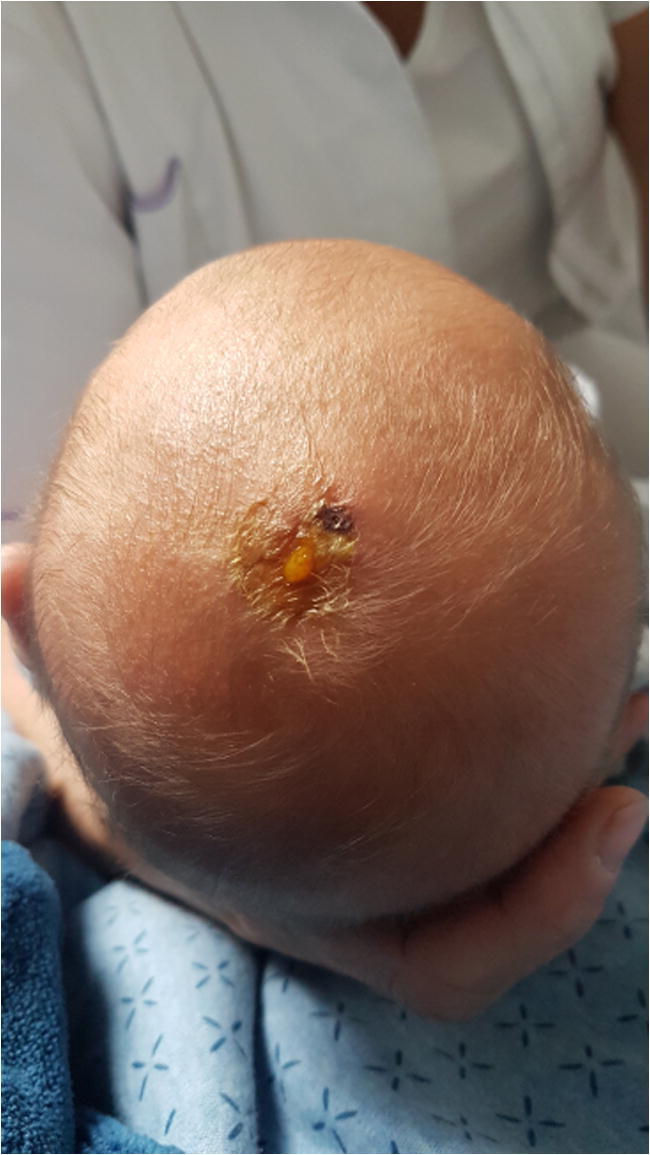
Five days postpartum superficial infection of the laceration left parasagittal where the fetal scalp electrode was placed during delivery

Our patient was given birth after 38+6/7 weeks of gestation (Apgar score 9/10). A fetal scalp electrode for cardiotocography was indicated for monitoring due to oligohydramnios. Birth length and weight measured 49cm (SDS −1.44) and 2.88kg (SDS −1.1), respectively. Head circumference was 34cm (SDS −1.25). The FSE left a small laceration which after a couple of days showed infection (Fig. 1). This was treated with fusidic acid cream 20mg/g for 5 days and led to a full recovery of the skin site.

**Fig. 2:**
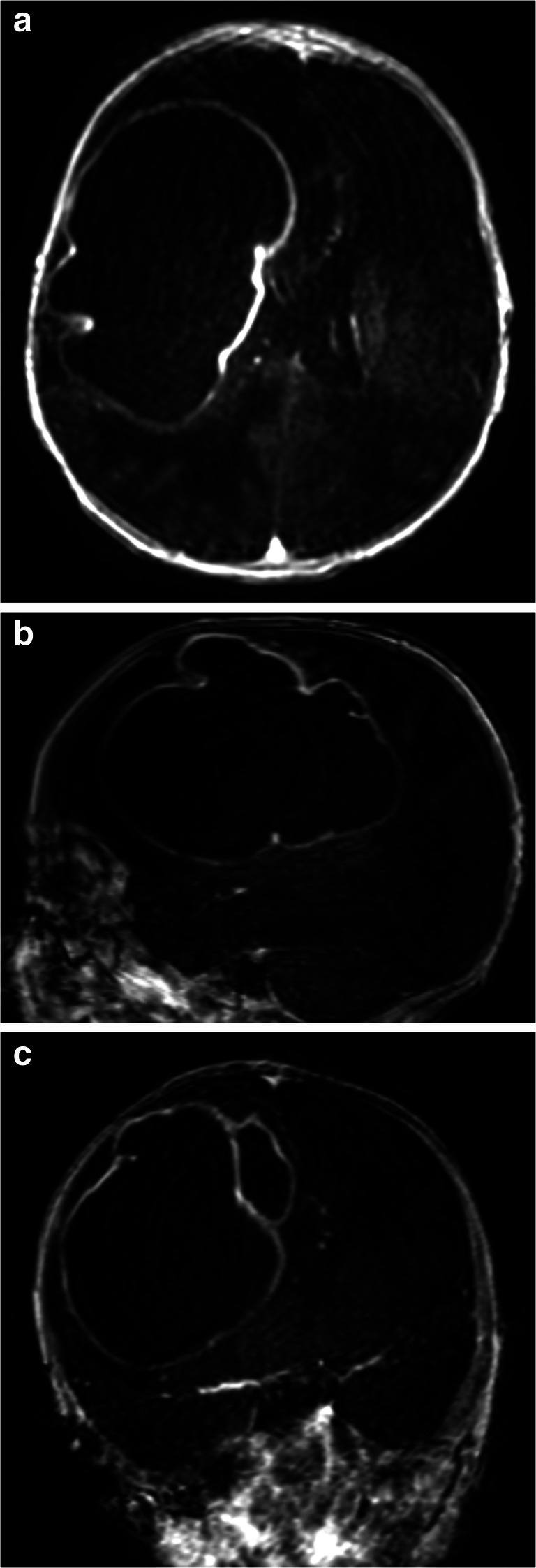
T1-weighted MRI with contrast showing a ring enhanced lesion in the right hemisphere with surrounding edema and midline shift. Mass dimensions: 78×51×58mm with an estimated volume of 120 ml. **a** Axial view. **b** Sagittal view. **c** Coronal view

At the infant consultation clinic at 8 weeks of age, an asymmetry of the skull was noticed. Body length and weight followed normal growth patterns, but head circumference revealed progressive head growth with crossing of percentiles (SDS +1.33). Although the child had no neurological deficits or signs of clinical illness, the fontanel was tensed and sutures were splayed prompting referral to our hospital for consultation and MRI imaging. The MRI showed a large mass lesion in the right hemisphere suspect for abscess based on the ring-enhanced aspect on the T1-weighted sequence with contrast and hyperintense with hypointense walls on T2 (Fig. 2). Blood work showed leukocytes of 26.4×10^9/l.

**Fig. 3:**
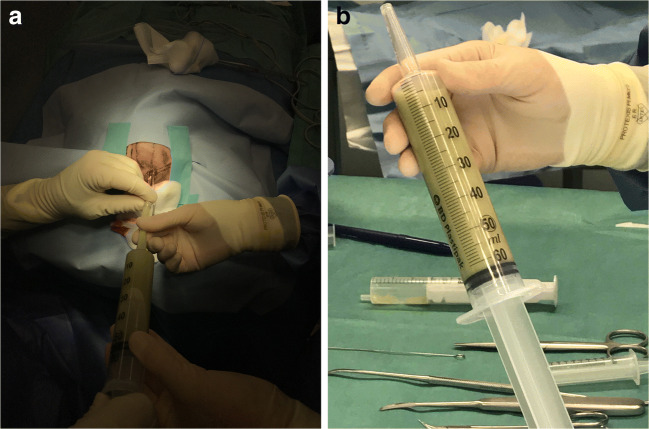
**a** Aspiration through right frontal incision. **b** Syringe showing purulent aspirate

Surgery was indicated, and 80 ml of green purulent fluid was aspirated in the OR using a blunt Dandy needle (Fig. 3a and b). We stopped surgery when aspiration was no longer possible due to resistance. Post aspiration ultrasound imaging in the OR revealed a small abscess residual.

Culture showed multiple pathogens, i.e., *Gardnerella vaginalis*, *Prevotella amnii*, and *Dialister micraerophilus* for which we initiated intravenous antibiotic treatment with ceftriaxone (100mg/kg/day in 1 dose) and metronidazole (30mg/kg/day in 3 doses). Postoperatively the patient remained clinically stable. Head circumference was measured daily, and ultrasound was planned 1 week after surgery. Ultrasound imaging showed significant increase of the remnant abscess. MRI imaging of the head confirmed the recurrence of the abscess with almost similar previous dimensions. Surgery was repeated, and this time 120 ml of purulent fluid could be aspirated. Culture revealed *Mycoplasma hominis* for which ciprofloxacin (30mg/kg/day in 2 doses) was added to the antibiotic treatment. Weekly ultrasound follow-up showed a stable volume of the remaining mass, and after 5 weeks, repeat MRI showed significant decrease (>90%) of the abscess volume.

After 6 weeks, the patient was discharged from the hospital in good clinical condition. Intravenous antibiotic treatment was continued for 8 weeks total after which ceftriaxone and metronidazole were stopped and ciprofloxacin was continued for another 2 weeks orally. At 2-year follow-up, our patient showed a slight developmental delay in gross motor skills. Further development was average for his age.

## Discussion

We presented a case-report of an infant developing a very large brain abscess following a superficial infection of the scalp due to an FSE placement. FSE is a widely used instrument during labor in clinical centers. Knowledge about the possible complications is therefore important. Brain abscess is a serious disease with a mortality rate in children of 3.7–12.5% [9–12]. Furthermore, morbidity rate is high with, after surgery, 29% of the patients suffering from epilepsy within the first month and up to 41.7–59% in the long term [2, 10, 13]. Moreover, brain abscess at young age is associated with mental retardation [10, 14].

To the best of our knowledge a cerebral abscess due to an FSE has been described only once before in literature [15]. The previously described case showed several similarities to our case. A deep laceration was noted after removal of the FSE. Microflora associated with the cervicovaginal region was cultured, and treatment consisted of wound cleansing and antibiotics for 7 days. After 6 weeks, an enlarged head circumference was noticed, and MRI imaging was performed. The abscess was treated with a one-time aspiration.

In both patients, the brain abscess was diagnosed after measuring an enlarged head circumference at the local child health clinic while both in clinically good condition without neurological deficits or fever. Since the development of the abscess can be clinically silent, follow-up is vital to intercept this illness in an early stage. Furthermore, late diagnosis and treatment is related to a higher mortality and morbidity rate [9, 16]. It is therefore recommended to organize an out-patient clinical follow-up for every infant with a superficial infection after placement of an FSE, which should comprise neurological examination and measurement of the head circumference. When abnormal findings are present, neuroimaging examination is strongly advised.

## Conclusion

A brain abscess in neonates is a possible life-threatening illness with a significant risk of developing epilepsy and mental retardation. These brain abscesses can develop progressively into very large volumes without signs of clinical illness. Therefore, screening and follow up of neonates after superficial infection due to an FSE is highly recommended.

## Data Availability

Not applicable.

## Abbreviations

CTG: Cardiotocography
FSE: Fetal scalp electrode
SDS: Standard deviation score
